# Locked Nucleic Acid Pentamers as Universal PCR Primers for Genomic DNA Amplification

**DOI:** 10.1371/journal.pone.0003701

**Published:** 2008-11-11

**Authors:** Zhen Sun, Zhi Chen, Xiaoli Hou, Shuping Li, Haihong Zhu, Ji Qian, Daru Lu, Wei Liu

**Affiliations:** 1 Department of Biochemistry and Molecular Biology, Zhejiang University School of Medicine, Hangzhou, Zhejiang, People's Republic of China; 2 The State Key Laboratory of Genetic Engineering and The MOE Key Laboratory of Contemporary and Anthropology, Fudan University School of Life Science, Shanghai, People's Republic of China; 3 National Key Laboratory for Diagnosis and Treatment of Infectious Disease, Institute of Infectious Disease, First Affiliated Hospital and Institute of Medical Biotechnology, Zhejiang University School of Medicine, Hangzhou, Zhejiang, People's Republic of China; Temasek Life Sciences Laboratory, Singapore

## Abstract

**Background:**

Multiplexing technologies, which allow for simultaneous detection of multiple nucleic acid sequences in a single reaction, can save a lot of time, cost and labor compared to traditional single reaction detection methods. However, the multiplexing method currently used requires precise handiwork and many complicated steps, making a new, simpler technique desirable. Oligonucleotides containing locked nucleic acid residues are an attractive tool because they have strong affinities for their complementary targets, they have been used to avoid dimer formation and mismatch hybridization and to enhance efficient priming. In this study, we aimed to investigate the use of locked nucleic acid pentamers for genomic DNA amplification and multiplex genotyping.

**Results:**

We designed locked nucleic acid pentamers as universal PCR primers for genomic DNA amplification. The locked nucleic acid pentamers were able to prime amplification of the selected sequences within the investigated genomes, and the resulting products were similar in length to those obtained by restriction digest. In Real Time PCR of genomic DNA from three bacterial species, locked nucleic acid pentamers showed high priming efficiencies. Data from bias tests demonstrated that locked nucleic acid pentamers have equal affinities for each of the six genes tested from the *Klebsiella pneumoniae* genome. Combined with suspension array genotyping, locked nucleic acid pentamer-based PCR amplification was able to identify a total of 15 strains, including 3 species of bacteria, by gene- and species-specific probes. Among the 32 species used in the assay, 28 species and 50 different genes were clearly identified using this method.

**Conclusion:**

As a novel genomic DNA amplification, the use of locked nucleic acid pentamers as universal primer pairs in conjunction with suspension array genotyping, allows for the identification of multiple distinct genes or species with a single amplification procedure. This demonstrates that locked nucleic acid pentamer-based PCR can be utilized extensively in pathogen identification.

## Introduction

The basic components of nucleic acid detection methodologies are the assay chemistry and the analysis platform. Well-characterized genotyping technologies include both solid phase (gels, DNA chips, glass slide arrays) and homogeneous solution (mass spectrometry, capillary electrophoresis) assay formats. Multiplexing technologies, which allow for simultaneous detection of multiple nucleic acid sequences in a single reaction, can greatly reduce the time, cost and labor spent compared to single reaction detection technologies. Recently, Sanchez and Endicott [1] reported a novel genotyping strategy in which two-stage multiplex PCR and capillary electrophoresis were used to simultaneously type all of the target sites. However, their procedure requires complicated steps. Furthermore, the multiplex PCR amplification procedure needs strict design and optimization, especially with regard to the primer sequences and the hybridization locations so as to prevent biased priming.

Previous research has suggested that oligonucleotides containing locked nucleic acid (LNA) residues have strong affinities for their complementary targets [2]. Locked nucleic acid pentamers are interspersed repetitively throughout the bacterial genome. The higher thermal stability and *T_m_* of DNA-LNA interactions as compared to DNA-DNA interactions could differentiate between matched and mismatched duplexes [3]–[5]. Therefore, LNA monomers have been widely accepted to avoid dimer formation and mismatch hybridization and to enhance efficient priming.

To explore the utility of LNA in multiplex PCR, we used interspersed locked nucleic acid pentamers (ILP) complementary to a sequence that appears repetitively in the bacterial genome as universal PCR primers together with the *Taq* polymerase Stoffel fragment for genomic DNA amplification. This new ILP-based PCR method (ILP-PCR) has proven to be efficient and stable, and only has rare priming biases. Our results suggest that combined with suspension arrays, ILP-PCR has the potential for expanded use in genotyping.

## Results

### ILP design and analysis

**Figure 1:**
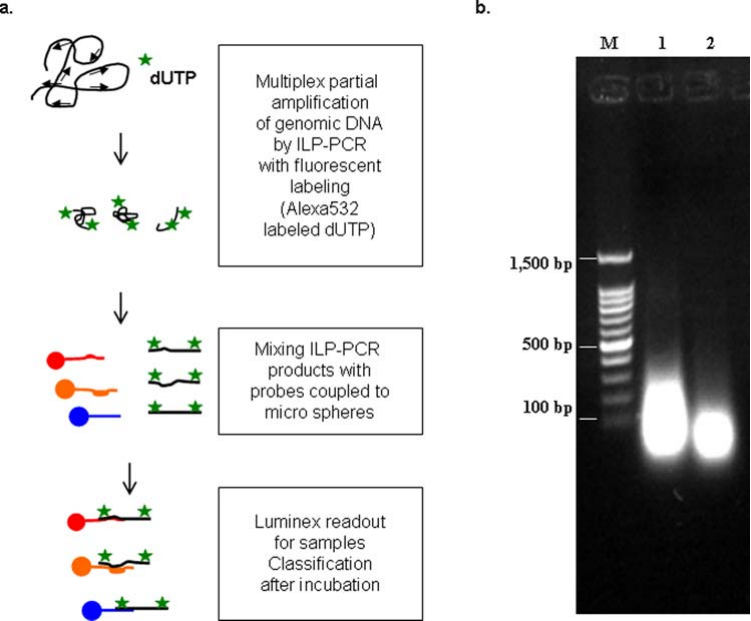
Whole genome amplification by interspersed LNA pentamers (ILPs). (a) Scheme of whole genomic DNA amplification by ILP-PCR in conjunction with the fluorescence embedding for suspension array genotyping. (b) Agarose gel electrophoresis of ILP-PCR products (1) and endonuclease (BsrSI+FokI) digestion products (2). The 100bp DNA ladder marker (M) was shown.

To explore the utility of LNA in multiplex PCR, we designed LNA primers against recognition sites of restriction endonucleases to be used as universal primers in a polymerase chain reaction. These LNA primers should be able to amplify genomic DNA starting at different specific sites, thereby “disassembling” the genomic DNA into short pieces. The PCR products could then be labeled with fluorescent nucleotides and subsequently used for suspension array-based genotyping (Figure 1a). When tested on bacterial genomic DNA, our ILPs primed selected recognition sequences within the investigated genomes and generated products of similar lengths to those obtained by digestion with restriction endonucleases (Figure 1b). The products of 80 to 250 bp are optimal for minimizing the potential of allelic drop-out [6], and are therefore suitable for array hybridization [7].

### Tracing ILP amplification kinetics

**Figure 2:**
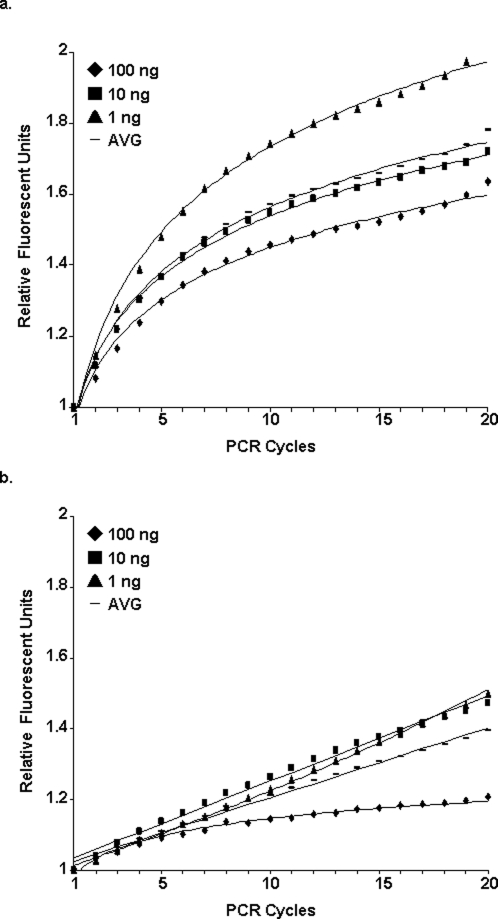
ILP amplification kinetics. Different concentrations of genomic DNA from 15 strains of 3 bacterial species were used for ILP-PCR (a) and random pentamer-PCR (b). The amplification signals at each cycle point and their average value (AVG) are shown.

Genomic DNAs from *Klebsiella pneumoniae* (ATCC700603 and twelve other isolates), *Klebsiella oxytoca* (one isolate) and *Escherichia coli* (ATCC25922), were digested by two restriction endonucleases (BsrSI and Fok I), and restriction products were separated on an agarose gel. We have designed two 5 bp ILPs, 5′-ACTGG-3′ and 5′-GGATG-3′ against the restriction sites. The priming efficiency of these ILPs, as compared to the efficiency of random pentamer primers (pd(N)_5_), was tested by a SYBR Green I-based real-time PCR reaction followed by fluorescent data analysis. Using input genomic DNAs (a mixture from all bacteria species) at various concentrations, the amplification plots of the ILP showed a stationary logarithmic curve (Figure 2a and Table S1) rather than the lines produced by the random pentamers (Figure 2b and Table S1). Additionally, final raw fluorescent signals (RFUs) generated by PCR with ILPs were significantly stronger than those generated using the random primers (1,200,000 RFUs vs. 320,000 RFUs, data not shown), and both were much higher than the negative control (NTC, 37,000 RFUs; data not shown). Unexpectedly, the highest priming efficiency was seen with 1 ng of input DNA and not with the 100 ng DNA sample (Figure 2a). The potential reason for this could be that excess DNA might lower the normal exposure of priming sites due to inefficient denaturation.

### Priming bias test

**Figure 3:**
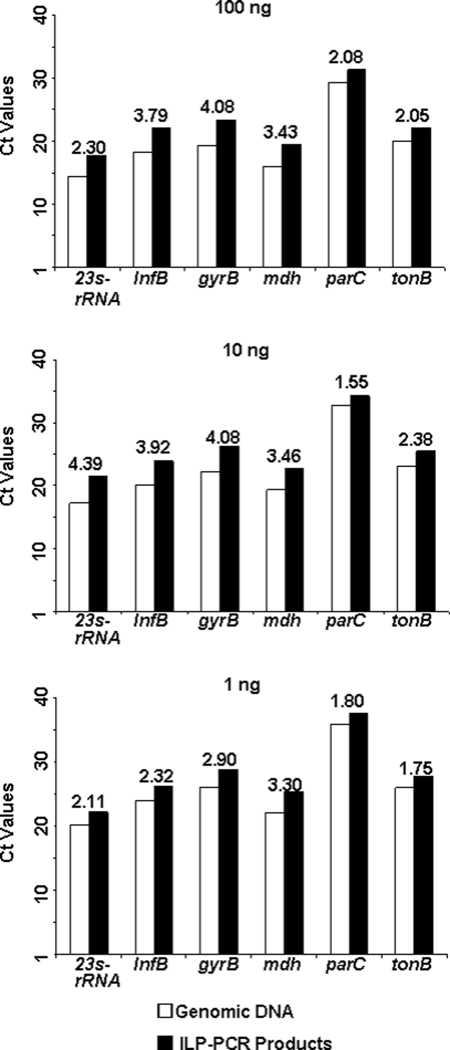
Test for biased priming during ILP-PCR. Six different genes from *K. pneumoniae* genomic DNA and the corresponding ILP-PCR products were selected for SYBR Green I real-time PCR analysis at different DNA concentrations. For all of the tested genes, the Ct value from the ILP-PCR product correlated well with that from the genomic DNA. The number above the bars showed the difference of each gene between the ILP-PCR product and the genomic DNA.

Six genes (*23S rRNA*, *infB*, *gyrB*, *mdh*, *parC* and *tonB*) in different loci of the *K. pneumoniae* genome were chosen (Table S2) to check for any biased priming when using ILPs. Bias was measured using real-time PCR followed by a SYBR Green I assay. Different amounts of bacterial genomic DNA (100 ng, 10 ng and 1 ng) and their corresponding ILP-PCR products were used for the bias test. As demonstrated in Figure 3, no false positive result was obtained by the dissociation test for any of the tested genes (Melting Curve Analysis, Figure S1). Separation by agarose electrophoresis yielded the same patterns as the expected size of the PCR products predicted by software (Figure S2). For each tested gene, the *Ct* values of the genomic DNAs at each concentration were similar to those for the ILP-PCR products (Figure 3).

### ILP-based suspension array genotyping and sensitivity testing

**Figure 4:**
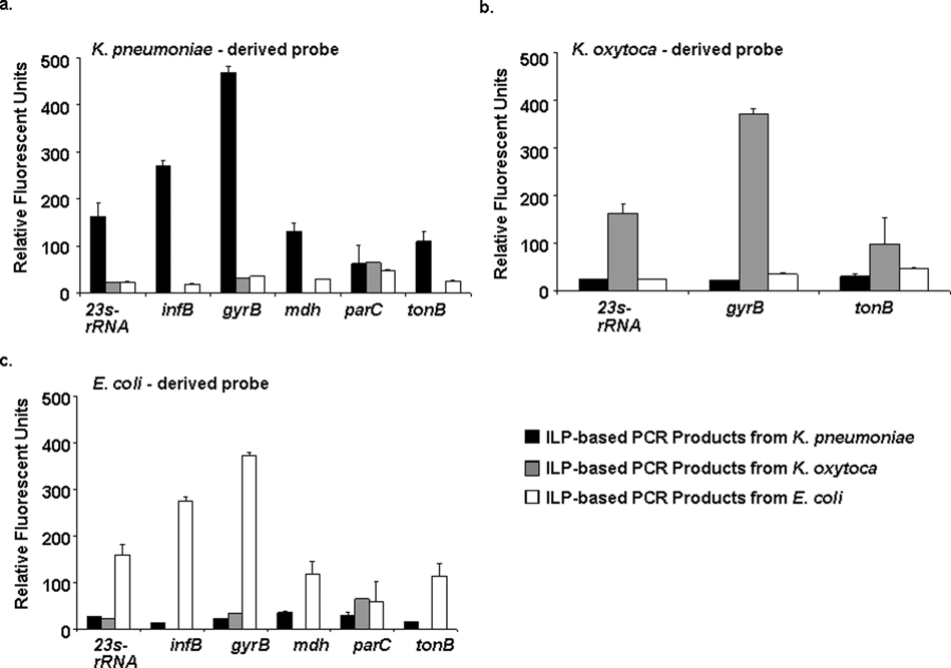
Cross-hybridization of gene- and species-specific probes and ILP-PCR products. Alexa Fluor® 532-labeled ILP-PCR products from 3 bacterial species were incubated with gene-specific probes from different bacterial origins for 20 minutes. The fluorescence signals were then scored using a suspension array flow analyzer. Results for six of the tested genes are shown. In some experiments, data from only 3 genes are presented due to the unavailability of the gene sequences.

A specific hybridization test was performed between the individual bacteria species and the probe sets (a total of fifteen tests). The genomic DNA mixture was amplified using ILPs. Alexa Fluor® 532-labeled dUTP was also included in the fragmentation procedure. After twenty cycles of amplification, the PCR products were fluorescent labeled, then they were incubated with the gene- and species-specific probe coupled to microspheres (Table S3). The signals were captured and read out with a suspension array flow analyzer. Of the tested bacterial species, including *K. pneumoniae* (13 identified/13 tested), *K. oxytoca* (1 identified/1 tested) and *E. coli* (1 identified/1 tested), all the samples were correctly identified. As shown in Figure 4, among the six genes tested, five genes were recognized specifically, where their mean fluorescence intensity was more than double the standard deviation (SD). Only *parC* was not recognized specifically. The results show that there was no cross hybridization or species mismatch (Figure 4).

### ILP-based high-throughput genotyping for multi–gene and -species identification

**Figure 5:**
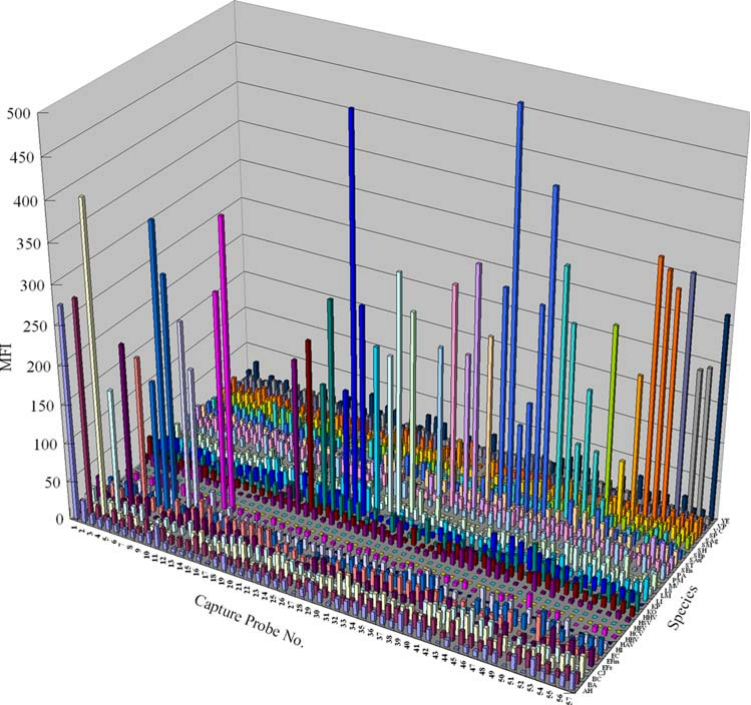
Multi-gene/species identification using ILP-based high throughput genotyping. A mixture of ILP-PCR products from 32 species (Y axis) were incubated with 57 specific capture probes (X axis) for 20 minutes, after which the fluorescence signals were scored using a suspension array flow analyzer. In the figure, each color indicates one species. For in detail knowledge about the probes and species, see supporting information Table S4. MFI: mean fluorescent intensity.

In order to determine the maximum potential of the ILP-based multiplex genotyping assay in gene and species identification, we tested 57 different genes from 32 distinct species of pathogenic microbes. We have previously reported some of the probes and positive controls for these 32 pathogens [8]–[10]. In this study, these probes were modified to include LNA monomers, using human genomic DNA as a negative control. As shown in Figure 5, after “disassembling” the mixed genomes with ILP, 28 of 32 tested pathogens were simultaneously detected. The four samples that were not detected were all RNA viruses (7 genes) whose ILP-PCR products were extremely unstable. The fluorescence signals of the 28 pathogenic samples, including 50 of 57 test genes, were much stronger than those of the negative controls (Figure 5). These results suggest that ILP-PCR-based amplification, in conjugation with suspension array genotyping, can identify at least 28 species or 50 genes with a single experiment.

## Discussion

Accurate and rapid identification of pathogens is crucial to clinical diagnosis and therapy, where this is also a critical part of epidemiological studies. Today, multiplexing is being used in the detection of bacterial [10]–[13], viral [8], [14] and fungal [15] pathogens. Probes or antibodies specific for environmental, food-borne and clinically relevant organisms could be easily added to this system, thus maximizing its efficiency [12]–[14], [16]–[19]. However, the use of degenerate primers as universal primers for amplification of a single gene from different genotypes or species might lower the PCR efficiency and increase non-specific amplifications [8], [10], [13]. Thus, for a multi-gene PCR assay, more steps using different primer pairs are needed to generate an ideal product for genotyping.

In this study, we designed ILPs to serve as universal primers for amplification of genomic DNA. The site-fixed ILPs have higher *T_m_* values and stronger affinities compared to normal DNA pentamers. Our ILPs displayed consistent logarithmic amplification plots, demonstrating a sustained amplification efficiency, while the random pentamers with more hybridization sites led to inconsistent plots. We used the *Taq* DNA polymerase Stoffel fragment instead of the DNA polymerase typically used for PCR amplification, because the former has a higher thermo-stability and lacks 5′ to 3′ exonuclease activity. In ILP-PCR, the use of the Stoffel fragment would facilitate the generation of smaller PCR products with enhanced reproducibility [20], [21]. In addition, the enhanced thermo-stability of the Stoffel fragment results in better efficiency when used with G+C rich templates or templates having complex secondary structure [22].

For any new whole-genome amplification method, it is important to test the possibility of biased priming. In order to evaluate the degree of bias in priming with ILP-PCR, we checked six genes in different loci of the *K. pneumoniae* genome by using SYBR Green I-based real-time PCR. Our results indicated clearly that the data from the ILP-PCR products were very similar to the values obtained from genomic template (GT) for of all the genes tested, suggesting that ILPs can be used efficiently and precisely for genomic DNA analysis.

In suspension arrays, it is important that the reporter should be selected carefully to avoid signal cross-contamination with classification fluorescence (635 nm). Generally, reporter fluorescence is excited by a 532 nm green laser, and the emission signal is recorded at 565–585 nm [23]. In this study, we chose Alexa Fluor® 532-labeled dUTP as a reporter, which has a 525 nm absorption peak and a 550 nm emission peak [24]. Our results demonstrated that, compared to other fluorophores, the small Alexa Fluor® 532 has much higher brightness, photo-stability and water solubility and can therefore be used directly in suspension arrays after ILP-PCR.

Generally, housekeeping genes give high signals when analyzed by suspension arrays. In our study, the *parC*-associated probes showed lower fluorescent signals and lower signal/noise ratios compared to the other genes analyzed. We think this might be the consequence of the low representation of the *parC* gene after the fragmentation procedure. After sequencing, we found that the tested *parC* gene sequences were commonly mutated in different species and even in different isolates of the same species (data not shown). This makes it difficult to design a universal capture probe to provide a high positive signal. Further, the sequence of the *parC* gene is very C/G rich, which causes more probe/template dimers or hairpins. Interestingly, the *gyrB* gene displayed a stronger signal than both the 23s-rRNA gene and the mdh gene even though the *gyrB* gene is present at a lower copy number in the genome. Sequence analysis demonstrated that the *gyrB* ILP-PCR product was more conserved, implying that multiple factors might be involved in the signal generation. Although highly conserved genes produce higher signals, this has disadvantages in classification. We therefore chose probes containing LNA residues in suspension arrays to elevate discrimination and maintain high gene signals.

Our new approach for amplifying and identifying multiple genes from different species is simpler and more efficient than other previously published methods [19], [25] It allows genotyping reactions to be carried out in one tube at the same time without any incubation or purification of specific reporter constructs. The assay comprises only three steps: genome amplification, probe hybridization and broad target identification. Carefully selected ILP sequences for diverse species would achieve high amplification, while a test for biased priming would assist in the design of genotyping probes. An easy way to design ILPs is to refer to the recognition sequences of restriction endonucleases.

In summary, we reported a novel ILP-based PCR method for genomic DNA amplification with unprecedented high efficiency. This technique holds great promise for routine microbial diagnostics in laboratories.

## Materials and Methods

ILPs were designed according to the recognition sites of the restriction endonuclease tested previously on the target genomic DNA (Each ILP should have a *T_m_* value between 40°C and 50°C, and its secondary structure and self-hybridization score should below 50. For LNA *T_m_* and spiked oligo hybridization tests, see http://lnatools.com for protocols). Usually, two or more ILPs could be used to generate products with different lengths. Probes for the suspension array typing assay were designed using a web-based software (http://lnatools.com). The ILPs and probes were synthesized and HPLC purified by IBA BioTAGnology (IBA GmbH, Göttingen, Germany). All probes were synthesized with an amino- and a carbon-linker modification at the 5′ terminus. Each probe was covalently coupled to carboxylated microspheres (QIAGEN) according to the manufacture's protocol. All primers used for testing the biased priming of ILPs were designed with Lasergene v6.1 software (DNAStar, Inc., Madison, WI, USA). They were designed against the conserved regions of bacterial genomes where no sequences of the applied ILPs could be found. The primers were synthesized by TaKaRa (TaKaRa, Dalian, China).

Sample DNAs from bacterial cultures (*E. coli, K. oxytoca, and K. pneumoniae*) were extracted and purified using DNeasy Blood & Tissue kit (QIAGEN). DNA concentrations quantified at OD_260nm/280nm_ on a BioPhotometer Plus (Hamburg, Germany) (1ng, 10ng and 100ng) to circumvent any source of error.

Genomic DNA mixtures were amplified using a 7500 Real-time PCR system (Applied Biosystems) followed by suspension array-based typing. Each 25 μL reaction contained 1×AmpliTaq Stoffel fragment polymerase and buffer (Applied Biosystems), 3 mM of MgCl_2_, 200 nM (each) of dATP, dCTP, dGTP (TaKaRa), 150 nM of Alexa Fluor 532-labeled dUTP (Molecular Probes), and 2.5 μM of each ILP . For monitoring of the ILP amplification kinetics, 1×SYBR Green I dye (Molecular Probes) and 1×ROX (Molecular Probes) were added, and 150 nM of dUTP purchased from TaKaRa was used. The cycling conditions were as follows: 5 min at 95°C; 20 cycles of 1 min at 95°C, 1 min at 40°C, 1 min at 50°C, and 1 min at 72°C; in 9600 emulation model, then, hold at 4°C.

The 20 μL reaction for the real-time PCR for the ILP priming bias test contained 1×SYBR-*Taq* mixture (Applied Biosystems), 0.2 μM of each primer, and 1 μL of each DNA template (genomic DNA or ILP products). The thermal cycling conditions were as follows: 95°C for 3min; 40 cycles of 95°C for 15 sec, 58°C for 10 sec, and 72°C for 40 sec; the fluorescence signals were read at the end of each cycle at 72°C . Dissociation test (Melting Curve) and agarose electrophoresis analysis were carried out after the 40 cycles were complete.

After a total of 20 cycles of ILP-PCR with the labeled dUTP, the products were mixed with 3,000 beads of each probe in a final volume of 50 μL. The mixture was incubated at 95°C for 5 min, followed by incubation at 50°C for 20 min. The mixture was then transferred to a 96-well filter plate. The beads were washed once and resuspended in SSC-Tween buffer. The fluorescent signals were detected according to the suspension array manufacturer's protocol.

## Supporting Information

Figure S1 The Melting Curve validation was performed to test the products from SYBR Green assays of six different gene loci. The three tested samples in this assay were randomly picked from the amplified real-time PCR products (A: *23S rRNA* products, B: *gyrB*, C: *infB*, D: *mdh*, E: *parC* and F: *tonB*). As the *tonB*-related product showed an unexpected sub-peak during this testing, agarose electrophoresis was carried out for further testing.
(0.17 MB DOC)

Figure S2 The products of the *tonB* gene-related SYBR Green I real-time PCR were separated on agarose gel. (M): 100bp DNA ladder. (Lanes 1-12): Real-time PCR products, which were amplified by using a series of diluted ILP-based PCR products (Dilution: 5-fold).
(0.07 MB DOC)

Table S1 The equations and R^2^ values of the trend lines from the plots of the ILP-PCR and random pentamer-based PCR.
(0.03 MB DOC)

Table S2 Primers used in the SYBR Green I-based real-time PCR with ILP-PCR products for the priming bias test.
(0.04 MB DOC)

Table S3 Probes used in the suspension array genotyping assay for the cross-hybridization/sensitivity test.
(0.05 MB DOC)

Table S4 Capture probes and sequences used for the multigene and -species identification assay.
(0.03 MB XLS)
