# Supplementary material for: Locked Nucleic Acid Pentamers as Universal PCR Primers for Genomic DNA Amplification
Source: PLoS One. 2008 Nov 11;3(11):e3701. doi: 10.1371/journal.pone.0003701 (PMC2577006; doi:10.1371/journal.pone.0003701)
Supplement: Figure S1 — The Melting Curve validation was performed to test the products from SYBR Green assays of six different gene loci. The three tested samples in this assay were randomly picked from the amplified real-time PCR products (A: 23S rRNA products, B: gyrB, C: infB, D: mdh, E: parC and F: tonB). As the tonB-related product showed an unexpected sub-peak during this testing, agarose electrophoresis was carried out for further testing. (0.17 MB DOC) [file pone.0003701.s001.doc]

**Supporting information, Figure S1**

Figure S1. The Melting Curve validation was performed to test the products from SYBR Green assays of six different gene loci. The three tested samples in this assay were randomly picked from the amplified real-time PCR products (A: *23S rRNA* products, B: *gyrB*, C: *infB*, D: *mdh*, E: *parC* and F: *tonB*). As the *tonB*-related product showed an unexpected sub-peak during this testing, agarose electrophoresis was carried out for further testing.


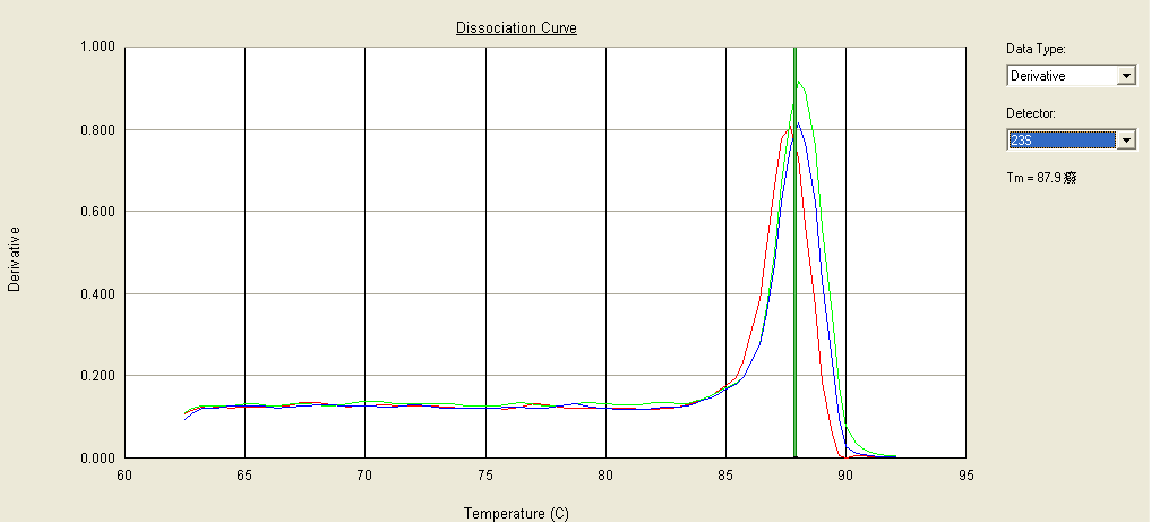


**A.**


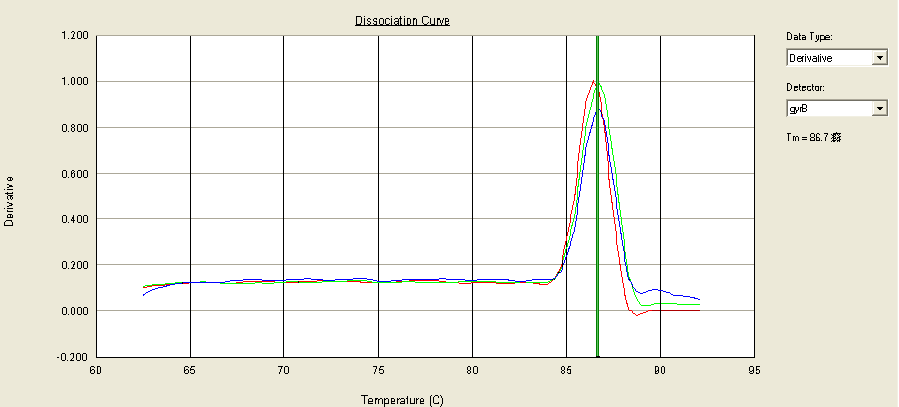


**B.**


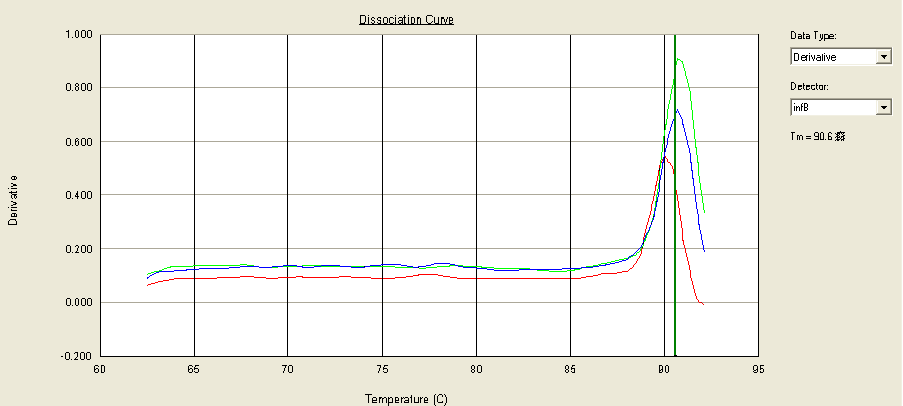


**C.**


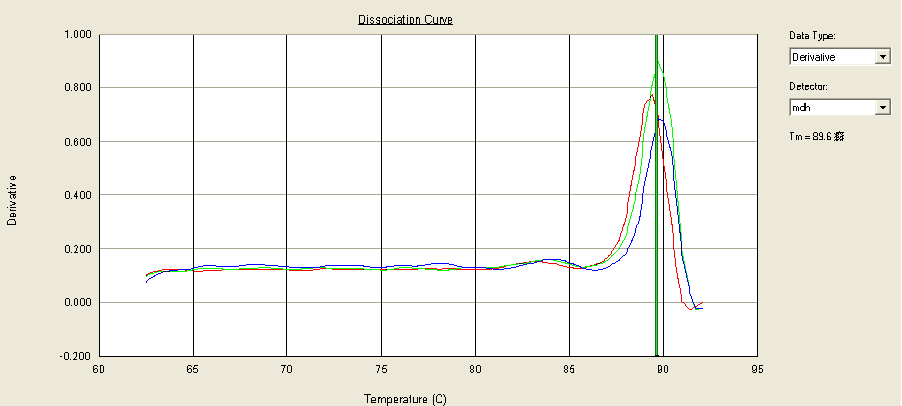


**D.**


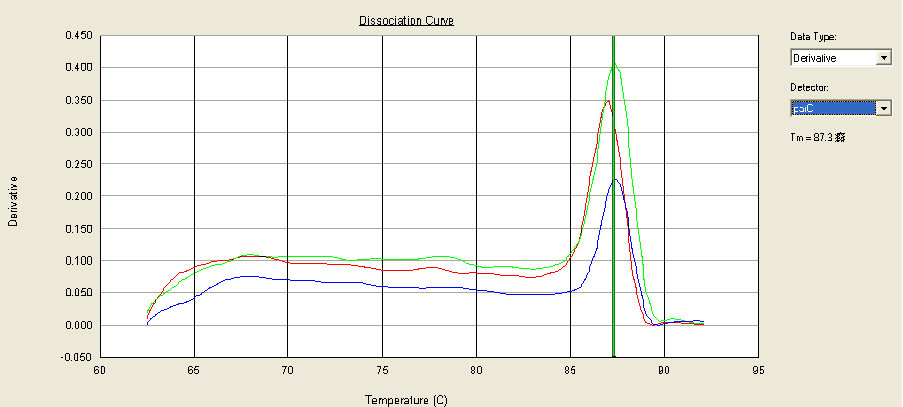


**E.**


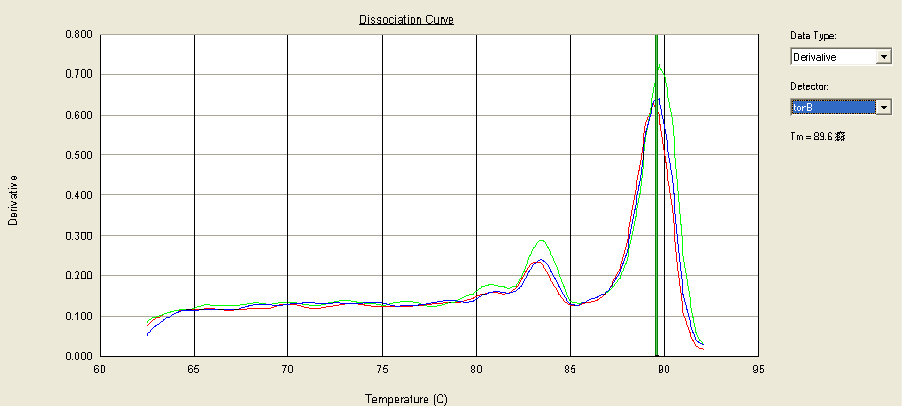


**F.**
