# Supplementary material for: Locked Nucleic Acid Pentamers as Universal PCR Primers for Genomic DNA Amplification
Source: PLoS One. 2008 Nov 11;3(11):e3701. doi: 10.1371/journal.pone.0003701 (PMC2577006; doi:10.1371/journal.pone.0003701)
Supplement: Figure S2 — The products of the tonB gene-related SYBR Green I real-time PCR were separated on agarose gel. (M): 100bp DNA ladder. (Lanes 1-12): Real-time PCR products, which were amplified by using a series of diluted ILP-based PCR products (Dilution: 5-fold). (0.07 MB DOC) [file pone.0003701.s002.doc]

**Supporting information, Figure S2**

Figure S2. The products of *tonB* gene related SYBR Green I real-time PCR were separated on agarose gel. (M): 100bp DNA ladder. (Lanes 1-12): Real-time PCR products, which were amplified by using a series of diluted ILP-based PCR products (Dilution: 5-fold).

**M 1 2 3 4 5 6 7 8 9 10 11 12**


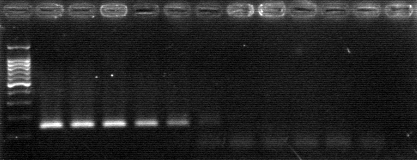


**2,000bp**

**200bp**

**100bp**

**Excess Primers**

**Unexpected peaks**
