# Supplementary material for: Locked Nucleic Acid Pentamers as Universal PCR Primers for Genomic DNA Amplification
Source: PLoS One. 2008 Nov 11;3(11):e3701. doi: 10.1371/journal.pone.0003701 (PMC2577006; doi:10.1371/journal.pone.0003701)
Supplement: Table S1 — The equations and R2 values of the trend lines from the plots of the ILP-PCR and random pentamer-based PCR. (0.03 MB DOC) [file pone.0003701.s003.doc]

Table S1. The equations and R^2^ values of the trend lines from the tracing plots of the ILP-PCR and random pentamer-based PCR.

| Input DNA | ILP-PCR | | Random pentamer-based PCR | |
| --- | --- | --- | --- | --- |
|  | equation | R^2^ | equation | R^2^ |
| 100 ng | Y = 0.2130Ln(x) + 0.9603 | 0.9900 | Y= 0.0706Ln(x) + 0.9836 | 0.9890 |
| 10 ng | Y = 0.2456Ln(x) + 0.9738 | 0.9962 | Y = 0.0238x + 1.0122 | 0.9898 |
| 1 ng | Y = 0.3465Ln(x) + 0.9368 | 0.9923 | Y= 0.9964e^0.0206x^ | 0.9978 |
| AVG | Y = 0.2633Ln(x) + 0.9581 | 0.9935 | Y = 0.0196x + 1.0083 | 0.9932 |
