# Supplementary material for: Locked Nucleic Acid Pentamers as Universal PCR Primers for Genomic DNA Amplification
Source: PLoS One. 2008 Nov 11;3(11):e3701. doi: 10.1371/journal.pone.0003701 (PMC2577006; doi:10.1371/journal.pone.0003701)
Supplement: Table S2 — Primers used in the SYBR Green I-based real-time PCR with ILP-PCR products for the priming bias test. (0.04 MB DOC) [file pone.0003701.s004.doc]

Table S2. Primers used in the Real-time PCR validation test of ILP-PCR products for priming bias test.

| Pathogens | Gene | Primer Sequences 5’-3’ | *Tm* (°C) | Size (bp) | GenBank Accession |
| --- | --- | --- | --- | --- | --- |
| *Klebsiella pneumoniae* | *23s-rRNA* | For-CAC GTG TCC CGC CCT ACT C  Rev-GAA CCG GGG GAA CTG AAA CA | 55.2  56.0 | 209 | X87284 |
|  | *infB* | For-CGC CGC GTT TAC CTC CAT  Rev-CGG ATC GGC TTC TGG CTT AT | 55.8  55.7 | 178 | AJ227990 |
|  | *gyrB* | For-GCG CGA CGG CAA AGA AGA  Rev-GGA AGC CGG CGA GGT GAG | 57.2  57.8 | 233 | AB084020 |
|  | *mdh* | For-GCG TGG CGG TAG ATC TAA GTC ATA  Rev-TTC AGC TCC GCC ACA AAG GTA | 56.4  57.1 | 364 | AM051124 |
|  | *parC* | For-CGG CGA CGT GTT GGG TAA ATA  Rev-CCA GCG GAT AGC GGT AAG AGA A | 57.2  57.3 | 98 | AF303625 |
|  | *tonB* | For-AGA GGC GCC GGT GGT GAT  Rev-CGG CGC CGT ATT GTT GTT TT | 57.5  57.3 | 160 | AF169814 |

“For”: forward primer, “Rev”: reverse primer.
