# Supplementary material for: Locked Nucleic Acid Pentamers as Universal PCR Primers for Genomic DNA Amplification
Source: PLoS One. 2008 Nov 11;3(11):e3701. doi: 10.1371/journal.pone.0003701 (PMC2577006; doi:10.1371/journal.pone.0003701)
Supplement: Table S3 — Probes used in the suspension array genotyping assay for the cross-hybridization/sensitivity test. (0.05 MB DOC) [file pone.0003701.s005.doc]

Table S3. Probes used in the suspension array genotyping assay in this study for cross-hybridization/sensitivity test.

| Pathogens | Gene | Probe Sequences 5’-3’ | GenBank Accession |
| --- | --- | --- | --- |
| *Klebsiella. pneumoniae* | *23s-rRNA* | NH_2_-C12-dA_15_-TCC CGT ACA C***C***A AAA ***T***GC AC | X87284 |
|  | *infB* | NH_2_-C12-dA_15_-GCC GCG TTT AC***C*** TC***C*** A | AJ227970 |
|  | *gyrB* | NH_2_-C12-dA_15_-CGA CGG CAA AGA AGA ***C***CA ***T***T | AB084020 |
|  | *mdh* | NH_2_-C12-dA_15_-TCC GAC CTG TTT AAT GT***G*** AA***T*** G | AJ635399 |
|  | *parC* | NH_2_-C12-dA_15_-CGC CAT CAG ***C***AC CAT ***C***G | AY034616 |
|  | *tonB* | NH_2_-C12-dA_15_-AGC C***C***G T***T***G T***T***G ***A***A | AF169816 |
| *Klebsiella. oxytoca* | *23s-rRNA* | NH_2_-C12-dA_15_-TCC CGT ACA C***T***A AAA ***C***GC AC | AF146763 |
|  | *infB* | No ideal probe available |  |
|  | *gyrB* | NH_2_-C12-dA_15_- CGA CGG CAA AGA AGA ***T***CA ***T***T | AB084018 |
|  | *mdh* | No enough sequence available |  |
|  | *parC* | NH_2_-C12-dA_15_-CGC CTG ***C***TA TGA AGC ***C***A | AF303647 |
|  | *tonB* | no sequence available |  |
| *Escherichia. coli* | *23s-rRNA* | NH_2_-C12-dA_15_-CCC CGT ACA C***A***A AAA ***T***GC AC | DQ682619 |
|  | *infB* | NH_2_-C12-dA_15_-CGC CGC GTT TAC ***T***TC ***A***A | AJ002408 |
|  | *gyrB* | NH_2_-C12-dA_15_-GAC GGC AAA GAA GA***C*** CA***C*** T | AY832967 |
|  | *mdh* | NH_2_-C12-dA_15_-CCG ACC TGT TTA ACG T***T***A A***C***G | DQ472587 |
|  | *parC* | NH_2_-C12-dA_15_-CTG ***T***TA TGA AGC ***G***AT GGT ***C***C | M58408 |
|  | *tonB* | NH_2_-C12-dA_15_-AGC C***G***G T***G***G T***A***G A***G***C | K00431 |

“NH_2”_” amino; “C12”: twelve carbons linker; “Bold and italic font”: LNA monomers.
